# ANGPTL2 overexpression promotes angiogenic responses in human retinal microvascular endothelial cells with accompanying integrin α5β1 molecular changes

**DOI:** 10.3389/fmed.2026.1846631

**Published:** 2026-08-03

**Authors:** Jiajia Song, Xiaofang Han, Jianfeng Chen, Tianrong Pan

**Affiliations:** 1 Department of Endocrinology, The Second Affiliated Hospital of Anhui Medical University, Hefei, China; 2 The Second People’s Hospital of Hefei, Hefei Hospital Affiliated to Anhui Medical University, Hefei, China

**Keywords:** angiopoietin-like protein, ANGPTL2, diabetic retinopathy, integrin α5β1, PI3K/AKT, retinal angiogenesis, retinal endothelial cells, streptozotocin

## Abstract

**Background:**

Diabetic retinopathy (DR) is a leading cause of preventable vision loss worldwide, characterized by retinal microvascular dysfunction and progressive angiogenic dysregulation. Angiopoietin-like protein 2 (ANGPTL2) promotes endothelial activation and pathological angiogenesis in multiple vascular disease contexts, yet its effects on retinal endothelial angiogenic behavior have not been directly characterized. Whether integrin α5β1 and PI3K/AKT phosphorylation changes in the diabetic retina are associated with alterations in ANGPTL2 expression levels also remains unexamined.

**Methods:**

ANGPTL2 was overexpressed in human retinal microvascular endothelial cells (HRMECs), and effects on cell viability, wound closure, invasion, and tube formation were assessed by CCK-8, wound healing, Transwell invasion, and tube formation assays. Integrin α5β1, VEGF, and PI3K/AKT phosphorylation were characterized by western blot. Retinal tissues from streptozotocin (STZ)-induced diabetic rats across groups with varying ANGPTL2 expression levels were analyzed by RT-qPCR, western blot, and immunofluorescence to characterize accompanying molecular changes. The protein-level association between ANGPTL2 and integrin α5β1 was examined by co-immunoprecipitation and co-localization immunofluorescence under overexpression conditions.

**Results:**

ANGPTL2 overexpression significantly enhanced cell viability, wound closure, invasion, and tube formation in HRMECs, accompanied by upregulation of VEGF, p-VEGFR2, integrin α5β1, and PI3K/AKT phosphorylation. Retinal tissues from STZ-diabetic rats showed elevated integrin α5β1 expression, increased CD31 levels, and increased PI3K/AKT phosphorylation relative to non-diabetic controls; these molecular changes were directionally lower in the ANGPTL2 knockdown comparison group (i.e., the STZ + sh-ANGPTL2 group), providing supportive retinal molecular context. Co-immunoprecipitation and co-localization analyses indicated a protein-level association between ANGPTL2 and integrin α5β1 under overexpression conditions.

**Conclusion:**

ANGPTL2 overexpression promotes angiogenic responses in retinal endothelial cells *in vitro*, with accompanying integrin α5β1 upregulation and broader signaling-associated phosphorylation changes. Retinal tissue molecular findings and the ANGPTL2–integrin α5β1 protein-level association provide supportive molecular context. The functional roles of specific molecular partners in mediating these responses remain to be determined through targeted validation studies.

## Introduction

Diabetic retinopathy is a major microvascular complication of diabetes mellitus and remains the leading cause of acquired vision loss among working-age adults globally (1). Chronic hyperglycemia drives retinal microvascular injury through mechanisms including endothelial dysfunction, pericyte loss, basement membrane thickening, and progressive breakdown of the blood-retinal barrier (2). As disease advances, dysregulated angiogenic signaling promotes the growth of fragile neovascular structures, a process central to vision-threatening stages of DR (3). Despite advances in anti-VEGF therapies and laser photocoagulation, a meaningful proportion of patients exhibit incomplete responses or disease recurrence, underscoring the need to identify additional molecular contributors to retinal vascular injury (4, 5).

Angiopoietin-like protein 2 (ANGPTL2), a secreted glycoprotein of the angiopoietin-like family, has emerged as a multifunctional regulator of vascular homeostasis and tissue inflammation (6). Originally characterized in adipose tissue and the cardiovascular system, ANGPTL2 promotes endothelial activation, leukocyte recruitment, and pathological angiogenesis in a range of metabolic and inflammatory contexts (7). Circulating ANGPTL2 levels are elevated in individuals with type 2 diabetes and correlate with systemic markers of vascular inflammation and microvascular dysfunction (8). Consistent with these systemic associations, serum ANGPTL2 rises progressively across the diabetic retinopathy severity spectrum—from non-proliferative to proliferative disease—and has been proposed as a prognostic marker and potential therapeutic target in diabetic retinopathy (9). At the cellular level, ANGPTL2 has been shown to enhance endothelial migration, sprouting, and tube formation—phenotypes directly relevant to pathological retinal angiogenesis (10). Despite this accumulating evidence, the functional consequences of ANGPTL2 dysregulation specifically within retinal endothelial cells remain to be directly addressed.

Integrin α5β1, a fibronectin-binding heterodimeric receptor expressed on endothelial cells, has been implicated in the regulation of retinal angiogenesis across multiple vascular disease models (11, 12). Its inhibition suppresses pathological neovascularization in experimental retinal models, in part through modulation of VEGF signaling and inflammatory cascades (13, 14). Importantly, ANGPTL2 has been reported to interact physically with integrin α5β1 and to promote integrin-dependent signaling in non-retinal cell systems (15, 16). The PI3K/AKT pathway, which operates downstream of integrin engagement and has been linked to endothelial cell survival, proliferation, and angiogenic activity in DR, represents a plausible molecular context in which ANGPTL2–integrin interactions may be relevant (17, 18). However, whether ANGPTL2 and integrin α5β1 are co-regulated in the diabetic retina, and whether their expression changes are accompanied by alterations in PI3K/AKT phosphorylation, has not been examined.

In the present study, ANGPTL2 was overexpressed in HRMECs to directly characterize its effects on retinal endothelial angiogenic behavior. An STZ-induced rat model of DR was used to provide retinal tissue molecular context, examining whether integrin α5β1 expression, CD31 levels, and PI3K/AKT phosphorylation changes in diabetic retinal tissues are directionally associated with ANGPTL2 expression levels. The protein-level association between ANGPTL2 and integrin α5β1 was further evaluated by co-immunoprecipitation.

## Materials and methods

The experimental modules, groups, sample sizes, assays and scope of inference for each figure are summarized in Table 1.

**Table 1:** Experimental design and assays for included figures.

Module	Group	Description	*n*	Assays	Scope of inference
A. HRMEC ANGPTL2 overexpression—functional (Figure 3)	Control/OE-NC/OE-ANGPTL2	Untransfected; empty vector; ANGPTL2 overexpression plasmid (Lipofectamine 2000; 48 h)	*n* = 3 independent experiments	RT-qPCR, WB (ANGPTL2); CCK-8; wound healing; Transwell invasion; tube formation	Primary functional evidence: ANGPTL2 overexpression associated with angiogenic phenotypes in HRMECs
B. HRMEC molecular markers (Figure 4)	Control/OE-NC/OE-ANGPTL2	Same HRMEC groups as Module A	*n* = 3 independent experiments	WB: VEGF, p-VEGFR2, VEGFR2, integrin α5, integrin β1, β-actin	Accompanying molecular marker changes noted; no functional blocking performed
C. HRMEC PI3K/AKT (Figure 5)	Control/OE-NC/OE-ANGPTL2	Same HRMEC groups as Module A	*n* = 3 independent experiments	WB: p-PI3K, PI3K, p-AKT, AKT, β-actin	Signaling-associated observation; no pharmacological inhibition; no pathway-level proof
D. Retinal tissue integrin α5β1 (Figure 1)	Control/STZ/STZ + sh-NC/STZ + sh-ANGPTL2	Non-diabetic controls; STZ-diabetic (12 wk); scrambled shRNA; ANGPTL2-targeting shRNA	6 animals/group; *n* = 3 biological replicates per assay (individual retinas)	RT-qPCR: integrin α5, β1; WB: integrin α5, β1; IF: integrin α5, β1	Supportive retinal molecular context; no functional integrin blocking; no *in vivo* causal relationship demonstrated
E. Retinal tissue PI3K/AKT (Figure 2)	Control/STZ/STZ + sh-NC/STZ + sh-ANGPTL2	Same retinal tissue groups as Module D	6 animals/group; *n* = 3 biological replicates per assay	WB: p-PI3K, PI3K, p-AKT, AKT, β-actin	Signaling-associated expression-level observation; no inhibitor experiments
F. ANGPTL2 in vivo expression (Supplementary Figure S2)	Control/STZ/STZ + sh-NC/STZ + sh-ANGPTL2	Same retinal tissue groups as Module D	*n* = 3 biological replicates per assay	RT-qPCR: ANGPTL2; WB: ANGPTL2, β-actin	Verification of ANGPTL2 expression levels and shRNA knockdown efficiency across groups
G. CD31 retinal expression (Supplementary Figure S3)	Control/STZ/STZ + sh-NC/STZ + sh-ANGPTL2	Same retinal tissue groups as Module D	*n* = 3 biological replicates per assay	WB: CD31, β-actin; IF: CD31	Supplementary vascular endothelial marker context; no direct functional assessment of vascular integrity
H. ANGPTL2–integrin α5β1 protein-level association (Supplementary Figure S1)	OE-ANGPTL2 HRMECs	ANGPTL2-overexpressing HRMECs; IgG control for Co-IP	Representative experiment	Co-IP (anti-ANGPTL2 pull-down, WB for integrin α5, β1); co-localization IF	Protein-level association under overexpression conditions only; endogenous interaction not assessed

### Animals and retinal tissue collection

Male Sprague–Dawley (SD) rats aged 7–10 weeks (Comparative Medicine Centre of Yangzhou University) were housed under standard conditions (22 ± 1 °C, 12 h light/dark cycle). Diabetes was induced by a single intraperitoneal injection of streptozotocin (STZ; 55 mg/kg; Bomeibio, Hefei, China) dissolved in citrate buffer (pH 4.5) following an overnight fast; controls received citrate buffer alone. Rats with fasting plasma glucose ≥ 16.7 mmol/L at day 7 post-injection were considered diabetic. Six animals were allocated to each group. Animals were sacrificed at 12 weeks post-induction and retinal tissues collected for molecular analyses. All procedures were approved by the Ethics Committee of The Second Affiliated Hospital of Anhui Medical University (IACUC-20230713) and conducted in accordance with ARRIVE guidelines.

**Figure 1:**
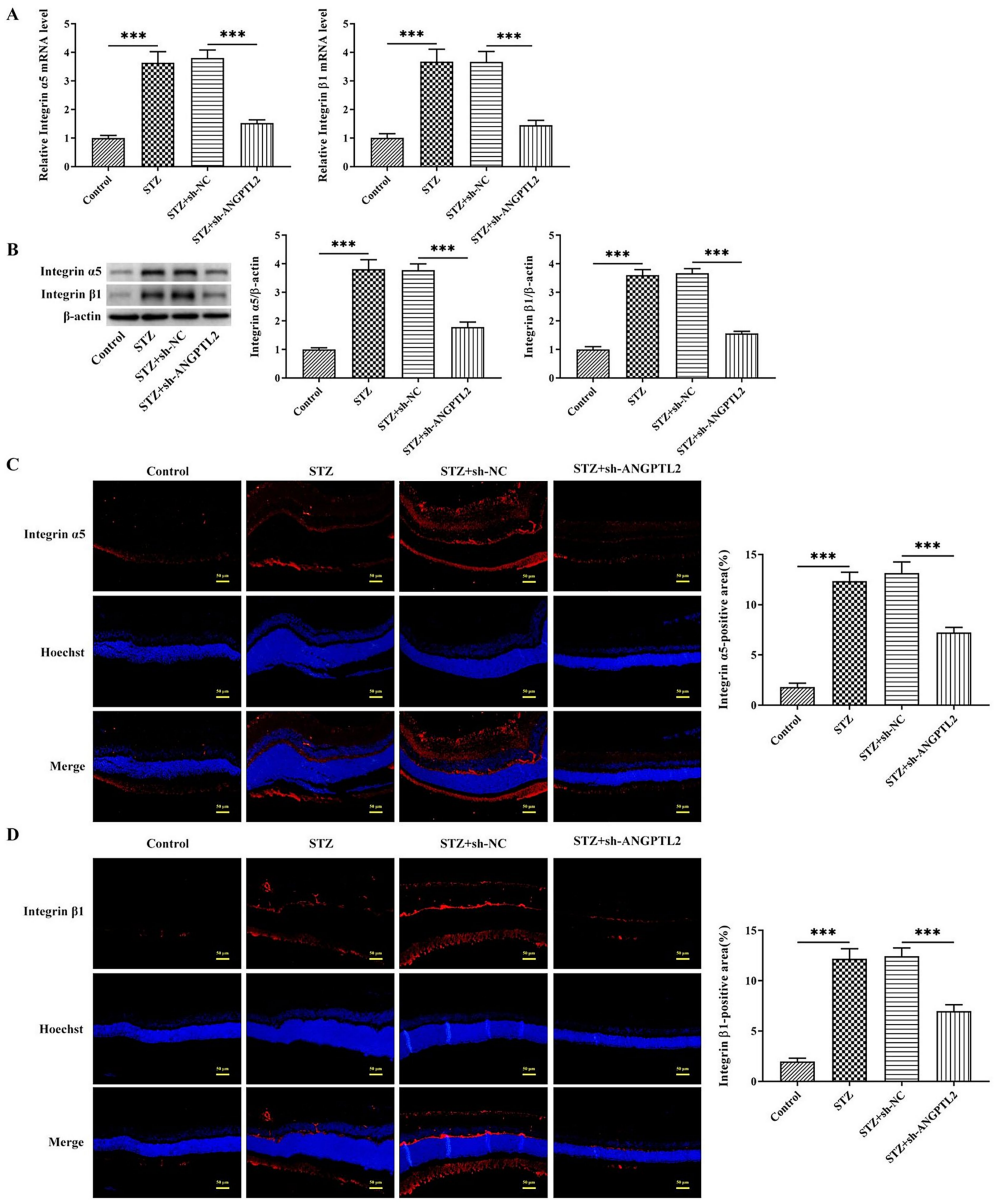
Integrin α5β1 expression is elevated in diabetic retinal tissues and shows a directionally lower pattern in the ANGPTL2 knockdown comparison group. **(A)** RT-qPCR analysis of integrin α5 and integrin β1 mRNA levels in retinal tissues from four groups (Control, STZ, STZ + sh-NC, STZ + sh-ANGPTL2), normalized to β-actin (*n* = 3 biological replicates per group, each representing an individual retina). **(B)** Representative western blot images and quantification of integrin α5 and integrin β1 protein expression normalized to β-actin (*n* = 3 biological replicates per group). Integrin α5: Control vs. STZ, *p* = 1.18 × 10^−6^; STZ + sh-NC vs. STZ + sh-ANGPTL2, *p* = 0.00002; STZ vs. STZ + sh-NC, ns. Integrin β1: Control vs. STZ, *p* = 7.72 × 10^−8^; STZ + sh-NC vs. STZ + sh-ANGPTL2, *p* = 4.00 × 10^−7^; STZ vs. STZ + sh-NC, ns. **(C)** Immunofluorescence staining for integrin α5 (red) with Hoechst 33342 nuclear counterstain (blue); merged images and quantification of integrin α5-positive area (%) from three separate animals (one representative section per animal; animal-level values used for statistical analysis) are shown. Scale bar, 50 μm. Control vs. STZ, *p* = 0.0002; STZ + sh-NC vs. STZ + sh-ANGPTL2, *p* = 0.0131; STZ vs. STZ + sh-NC, ns. **(D)** Immunofluorescence staining for integrin β1 (red) with Hoechst 33342 nuclear counterstain (blue); merged images and quantification of integrin β1-positive area (%) from three separate animals (one representative section per animal; animal-level values used for statistical analysis) are shown. Scale bar, 50 μm. Control vs. STZ, *p* = 1.55 × 10^−15^; STZ + sh-NC vs. STZ + sh-ANGPTL2, *p* = 2.44 × 10^−15^; STZ vs. STZ + sh-NC, ns. Data are presented as mean ± SD.

**Figure 2:**
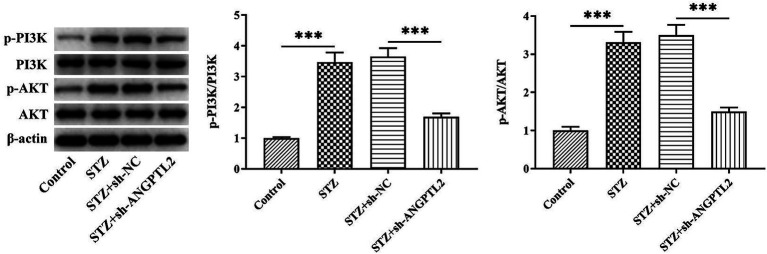
PI3K/AKT phosphorylation changes in diabetic retinal tissues are associated with ANGPTL2 expression levels across comparison groups. Representative western blot images showing p-PI3K, total PI3K, p-AKT, total AKT, and β-actin in retinal tissues from four groups (Control, STZ, STZ + sh-NC, STZ + sh-ANGPTL2). Quantification of p-PI3K/PI3K and p-AKT/AKT ratios (*n* = 3 biological replicates per group, each representing an individual retina) is shown. p-PI3K/PI3K: Control vs. STZ, *p* = 1.49 × 10^−6^; STZ + sh-NC vs. STZ + sh-ANGPTL2, *p* = 0.00001; STZ vs. STZ + sh-NC, ns. p-AKT/AKT: Control vs. STZ, *p* = 0.0006; STZ + sh-NC vs. STZ + sh-ANGPTL2, *p* = 0.0010; STZ vs. STZ + sh-NC, ns. Data are presented as mean ± SD.

Retinal tissues used for expression analyses in Figures 1 and 2, and Supplementary Figures S2 and S3 were obtained from four groups: non-diabetic controls (Control), STZ-diabetic rats without intravitreal injection (STZ), STZ-diabetic rats with intravitreal scrambled lentiviral shRNA (STZ + sh-NC), and STZ-diabetic rats with intravitreal ANGPTL2-targeting shRNA (STZ + sh-ANGPTL2). Intravitreal injections of lentiviral particles (1 × 10^9^ TU/mL; Obiosh, Shanghai; 2 μL per eye) were administered to both eyes at 4 and 8 weeks after STZ injection. One eye per animal was randomly assigned for retinal tissue collection for molecular analyses. Although six retinas were collected per group, each molecular assay was performed on three randomly selected biological replicates (*n* = 3; one retina per animal, each obtained from a separate animal), and the remaining retinas were retained as biological reserves and were not included in the reported quantifications. The cohort of six animals per group was planned *a priori* to accommodate the variable success of streptozotocin-induced diabetes and attrition in the diabetic groups while maintaining balanced group sizes across conditions. The shRNA target sequences are listed in Supplementary Table S1. ANGPTL2 expression levels in the four groups were confirmed by RT-qPCR and western blot (Supplementary Figure S2).

### Immunofluorescence staining

Retinal tissue sections were fixed in 4% paraformaldehyde (Sigma, MKCL5723), permeabilized with 0.1% Triton X-100 (BioFROXX, 1139ML500), and blocked with 5% BSA. Primary antibodies against integrin α5 (1:200; Proteintech, 10569-1-AP), integrin β1 (1:200; Proteintech, 26918-1-AP), or CD31 (1:200; Proteintech, 28083-1-AP) were applied overnight at 4 °C, followed by Alexa Fluor-conjugated secondary antibodies and Hoechst 33342 nuclear counterstain (Sigma, H6024). For HRMEC co-localization experiments, OE-ANGPTL2-transfected cells were stained with antibodies against ANGPTL2 (1:200; Abcam, ab113263), integrin α5, and integrin β1 as above. Images were quantified using ImageJ. Image quantification was performed without blinding to group assignment.

### RT-qPCR

Total RNA was extracted using TRIzol (Biosharp, BS259A) and reverse-transcribed with RevertAid cDNA Synthesis Kit (KCD-M1004). Quantitative PCR was performed on a LightCycler 480 II (Roche) with SYBR Green Master Mix; relative expression was calculated by the 2^−^ΔΔCt method with β-actin as reference. Primer sequences are listed in Supplementary Table S2.

### Western blot

Total protein was extracted in RIPA buffer and quantified by BCA assay (Biosharp, BL521A). Equal protein amounts were resolved by SDS-PAGE, transferred to PVDF membranes (Millipore, HATF00010), and blocked with 5% BSA for 2 h. Primary antibodies used: integrin α5 (1:1000; Proteintech, 10569-1-AP), integrin β1 (1:500; Proteintech, 26918-1-AP), p-PI3K (1:500; Affinity, AF3241), PI3K (1:500; Affinity, AF6241), p-AKT (1:500; Affinity, AF0016), AKT (1:500; Affinity, AF6261), ANGPTL2 (1:500; Proteintech, 12316-1-AP), VEGF (1:500; Proteintech, 19003-1-AP), total VEGFR2 (1:500; Affinity, AF6281), p-VEGFR2 Tyr1059 (1:500; Affinity, AF3279), CD31 (1:1000; Proteintech, 28083-1-AP), and β-actin (1:2000; Servicebio, GB11001). Bands were visualized by ECL reagent (Millipore, WBKLS0100) on a ChemiDoc XRS+ and quantified using ImageJ.

### Cell culture and ANGPTL2 overexpression

HRMECs (iCell Bioscience, HUM-CELL-0138) were maintained in endothelial cell medium (ScienCell, 1001) supplemented with 10% FBS and 1% antibiotic solution at 37 °C in 5% CO₂. ANGPTL2 overexpression plasmid (OE-ANGPTL2) and empty vector control (OE-NC) were transfected using Lipofectamine 2000 (Invitrogen, 11668-027). Cells were used 48 h post-transfection; overexpression was confirmed by RT-qPCR and western blot.

### Functional assays

CCK-8 cell viability assay: HRMECs were seeded at 5 × 10^3^ cells/well in 96-well plates; CCK-8 reagent (Biosharp, BS350B; 15 μL/well) was added after 24 h and absorbance measured at 450 nm after 2 h.

Wound healing assay: A uniform scratch was created in confluent monolayers using a 200 μL pipette tip; wound closure was quantified with ImageJ at 0 h and 48 h.

Transwell invasion assay: HRMECs were seeded in the upper chamber of Matrigel-coated Transwell inserts (8 μm pore; Corning) with medium containing 20% FBS in the lower chamber. After 24 h, invading cells were fixed, stained with 0.1% crystal violet (Beyotime, C0121), and counted in five fields per well.

Tube formation assay: Growth factor-reduced Matrigel (Corning) was polymerized in 6-well plates (37 °C, 30 min); HRMECs were seeded on the surface and cultured for 8 h; tube structures were counted in five fields per well. All functional assays were performed in three independent experiments.

### Co-immunoprecipitation

OE-ANGPTL2-transfected HRMECs were lysed 48 h post-transfection in Co-IP lysis buffer (Thermo Scientific, 87787). Cleared supernatants were incubated overnight at 4 °C with 5 μg anti-ANGPTL2 antibody or normal IgG control, followed by Protein A/G agarose beads (Bimake, B23201) for 2 h. Precipitates were analyzed by western blot for integrin α5 and integrin β1. A representative result is shown. Both Co-IP and co-localization assays were performed under ANGPTL2 overexpression conditions; endogenous interaction was not assessed.

### Statistical analysis

All data are expressed as mean ± SD. Normality was assessed by Shapiro–Wilk test. Two-group comparisons used unpaired Student’s *t*-test or Mann–Whitney *U* test as appropriate; three or more groups were analyzed by one-way ANOVA with Tukey’s *post hoc* test or Kruskal-Wallis test with Dunn’s comparison. Statistical analyses were performed using Python (SciPy v1.11) and verified in GraphPad Prism version 10.1.2. Exact *p* values are reported for all comparisons; where *p* < 1 × 10^−15^, *p* < 1e−15 is indicated. *p* < 0.05 was considered statistically significant. For *in vitro* experiments, *n* = 3 represents three independently performed experiments; for functional assays (CCK-8, wound healing, Transwell, tube formation), data represent three independent experiments with three replicates each (*n* = 9 per group). For retinal tissue molecular analyses, six retinas were collected per group; *n* = 3 biological replicates per assay represents three individual retinas from separate animals, as indicated in the corresponding figure legends. For each retinal molecular assay, three individual retinal samples, each obtained from a separate animal (one eye per animal), were analyzed as biological replicates.

## Results

### ANGPTL2 overexpression enhances angiogenic responses in HRMECs

**Figure 3:**
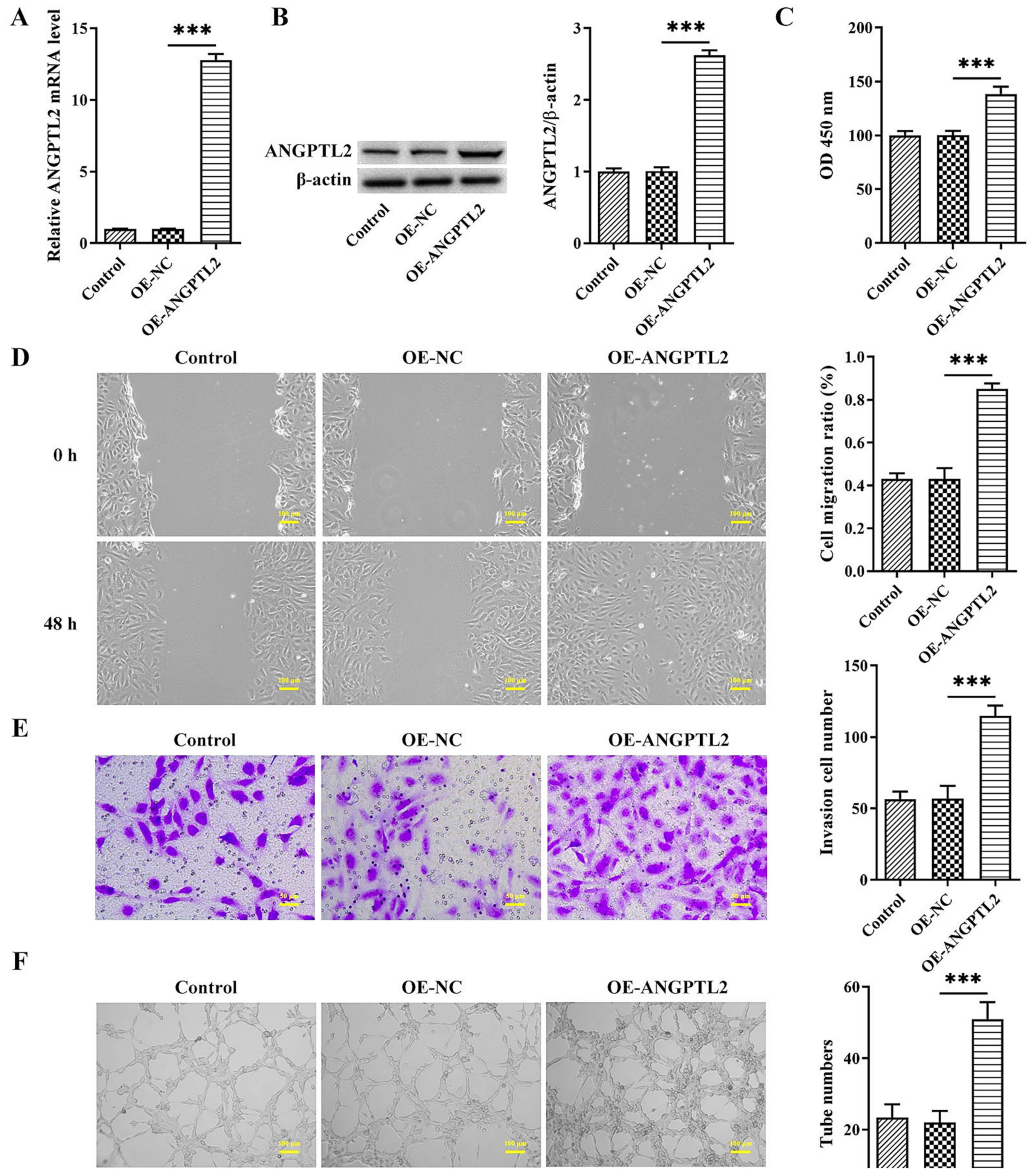
ANGPTL2 overexpression enhances angiogenic responses in HRMECs. **(A)** RT-qPCR of ANGPTL2 mRNA in Control, OE-NC, and OE-ANGPTL2 HRMECs, normalized to β-actin (*n* = 3 independent experiments). OE-NC vs. OE-ANGPTL2, *p* < 1 × 10^−15^. **(B)** Western blot and quantification of ANGPTL2 protein (*n* = 3 independent experiments). OE-NC vs. OE-ANGPTL2, *p* = 9.37 × 10^−8^. **(C)** CCK-8 cell viability assay (*n* = 3 independent experiments, 9 replicates per group). OE-NC vs. OE-ANGPTL2, *p* = 1.00 × 10^−13^. **(D)** Representative wound healing images at 0 h and 48 h with quantification of wound closure ratio (%) (*n* = 3 independent experiments, 9 replicates per group). Scale bar, 100 μm. OE-NC vs. OE-ANGPTL2, *p* < 1 × 10^−15^. **(E)** Representative Transwell invasion images with quantification of invading cell number per field (*n* = 3 independent experiments, 9 replicates per group). Scale bar, 50 μm. OE-NC vs. OE-ANGPTL2, *p* = 2.20 × 10^−14^. **(F)** Representative tube formation images with quantification of tube number (*n* = 3 independent experiments, 9 replicates per group). Scale bar, 100 μm. OE-NC vs. OE-ANGPTL2, *p* = 1.31 × 10^−13^. Data are presented as mean ± SD.

To directly assess whether ANGPTL2 influences retinal endothelial angiogenic behavior, ANGPTL2 was overexpressed in HRMECs. Transfection efficiency was confirmed by RT-qPCR (approximately 12-fold increase over OE-NC; Figure 3A) and western blot (Figure 3B).

CCK-8 assay demonstrated significantly increased cell viability in OE-ANGPTL2 cells relative to OE-NC, with no difference between OE-NC and untransfected controls (Figure 3C). Wound healing assays showed markedly enhanced wound closure at 48 h in OE-ANGPTL2 cells (Figure 3D). Transwell invasion assays showed a significant increase in invading cell numbers in the OE-ANGPTL2 group relative to OE-NC (Figure 3E). Tube formation assays showed that OE-ANGPTL2 cells generated approximately twice as many tubular structures as OE-NC cells (Figure 3F). These concordant multi-assay functional changes indicate that ANGPTL2 overexpression is sufficient to enhance angiogenic responses in HRMECs under these experimental conditions and constitute the primary functional contribution of this study.

### ANGPTL2 overexpression is accompanied by upregulation of angiogenic markers, integrin α5β1, and PI3K/AKT phosphorylation in HRMECs

**Figure 4:**
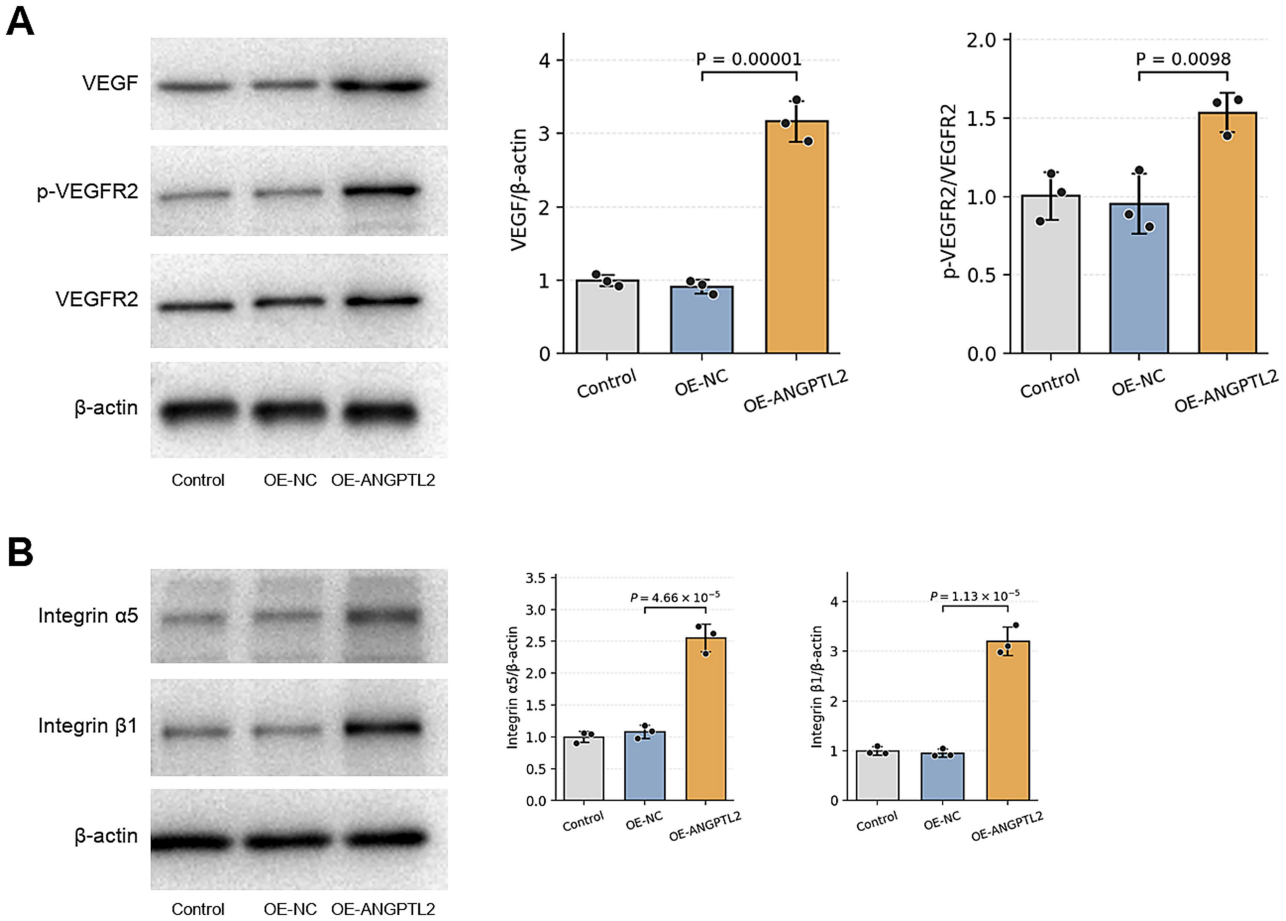
ANGPTL2 overexpression is accompanied by upregulation of angiogenic markers and integrin α5β1 in HRMECs. **(A)** Western blot of VEGF, p-VEGFR2 (Tyr1059), and total VEGFR2 with quantification (*n* = 3 independent experiments). VEGF/β-actin: OE-NC vs. OE-ANGPTL2, *p* = 0.00001. p-VEGFR2/VEGFR2: OE-NC vs. OE-ANGPTL2, *p* = 0.0098. **(B)** Western blot of integrin α5 and integrin β1 protein levels in Control, OE-NC, and OE-ANGPTL2 HRMECs with quantification (*n* = 3 independent experiments). Integrin α5: OE-NC vs. OE-ANGPTL2, *p* = 4.66 × 10^−5^ (one-way ANOVA with Tukey *post hoc*). Integrin β1: OE-NC vs. OE-ANGPTL2, *p* = 1.13 × 10^−5^ (one-way ANOVA with Tukey post hoc). Data are presented as mean ± SD.

Western blot analysis showed that VEGF and p-VEGFR2 (Tyr1059) levels were significantly elevated in OE-ANGPTL2 cells, while total VEGFR2 showed no significant change, consistent with upregulation of angiogenic signaling activity (Figure 4A). Integrin α5 and integrin β1 protein levels were also elevated in OE-ANGPTL2 cells relative to controls (Figure 4B), consistent with the retinal tissue molecular observations. This co-upregulation of integrin α5β1 with the angiogenic phenotype is noted as a molecular correlate; whether integrin α5β1 functionally mediates these responses remains to be determined in targeted validation studies.

**Figure 5:**
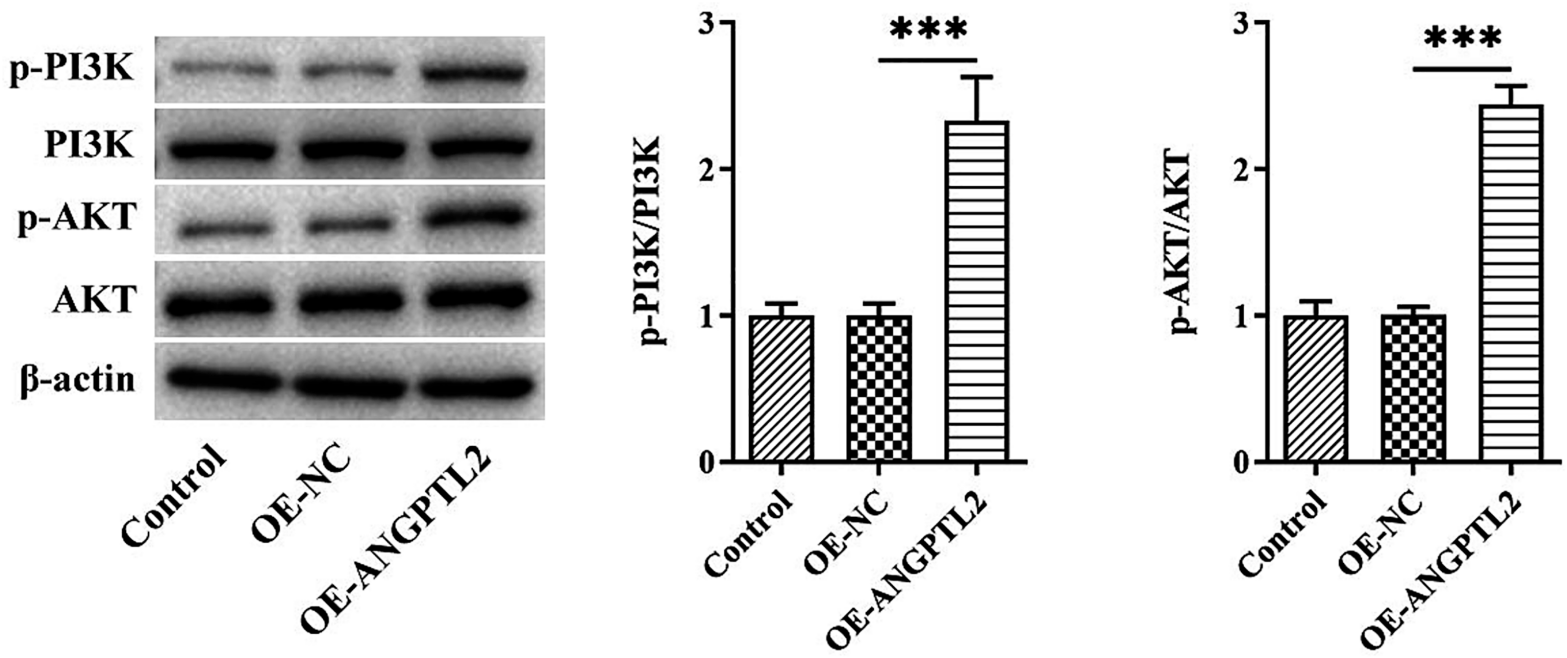
ANGPTL2 overexpression is accompanied by PI3K/AKT phosphorylation changes in HRMECs. Representative western blot images of p-PI3K, total PI3K, p-AKT, total AKT, and β-actin in Control, OE-NC, and OE-ANGPTL2 HRMECs. Quantification of p-PI3K/PI3K and p-AKT/AKT ratios (*n* = 3 independent experiments) is shown. No significant difference was detected between Control and OE-NC groups. p-PI3K/PI3K: OE-NC vs. OE-ANGPTL2, *p* = 0.00061. p-AKT/AKT: OE-NC vs. OE-ANGPTL2, *p* = 0.00006. ns, not significant (Control vs. OE-NC). Data are presented as mean ± SD.

To characterize the signaling context accompanying ANGPTL2-induced angiogenic responses, PI3K and AKT phosphorylation were assessed. p-PI3K/PI3K and p-AKT/AKT ratios were significantly higher in OE-ANGPTL2 cells than in OE-NC controls, while no difference was detected between untransfected controls and OE-NC (Figure 5). Total PI3K and AKT levels were comparable across groups. These phosphorylation changes are consistent with broad angiogenic activation accompanying ANGPTL2 overexpression. No pharmacological inhibition of PI3K or AKT was performed; these are signaling-associated observations that do not demonstrate PI3K/AKT as a validated downstream effector of ANGPTL2 in this model.

### Integrin α5β1 expression is elevated in diabetic retinal tissues and shows a directionally lower pattern in the ANGPTL2 knockdown comparison group

To provide retinal tissue molecular context for the *in vitro* findings, integrin α5β1 expression was examined across four groups that differ in their ANGPTL2 levels: non-diabetic controls (Control), STZ-diabetic rats (STZ), STZ-diabetic rats receiving scrambled intravitreal lentiviral shRNA (STZ + sh-NC), and STZ-diabetic rats receiving ANGPTL2-targeting lentiviral shRNA (STZ + sh-ANGPTL2). ANGPTL2 expression in these four groups was confirmed by RT-qPCR and western blot (Supplementary Figure S2). These comparisons were designed to characterize molecular co-variation across groups with differing ANGPTL2 contexts; differences between diabetic and non-diabetic groups reflect a complex hyperglycemic background in which multiple factors may contribute independently to observed molecular changes.

RT-qPCR showed that integrin α5 and integrin β1 mRNA levels were significantly elevated in STZ-treated animals relative to non-diabetic controls (Figure 1A). No significant difference was detected between STZ and STZ + sh-NC groups, indicating that lentiviral delivery per se did not alter integrin expression. In the STZ + sh-ANGPTL2 group, both mRNA levels showed a directionally lower pattern relative to STZ + sh-NC, consistent with reduced ANGPTL2 expression in that group. Western blot analysis showed directionally consistent protein-level patterns (Figure 1B). Immunofluorescence staining of retinal sections was consistent with the molecular data (Figures 1C,D). Collectively, these findings provide supportive retinal molecular context showing that integrin α5β1 expression co-varies with ANGPTL2 levels across comparison groups.

### PI3K/AKT phosphorylation changes in diabetic retinal tissues are associated with ANGPTL2 expression levels across comparison groups

Western blot analysis of retinal lysates showed that p-PI3K/PI3K and p-AKT/AKT ratios were significantly elevated in STZ animals compared with non-diabetic controls (Figure 2). No significant difference was detected between STZ and STZ + sh-NC groups. In the STZ + sh-ANGPTL2 group, both ratios were directionally lower than in STZ + sh-NC controls, in a pattern consistent with the lower ANGPTL2 expression levels in that group. Total PI3K and AKT levels were comparable across groups. These retinal tissue findings are consistent with the PI3K/AKT phosphorylation changes observed in OE-ANGPTL2 HRMECs (Figure 5) and provide supportive molecular context. As with integrin α5β1, hyperglycemia and associated pathological processes are independently capable of modulating PI3K/AKT phosphorylation, and the current data cannot attribute these changes specifically to ANGPTL2.

### CD31 expression in diabetic retinal tissues shows a pattern consistent with ANGPTL2-associated vascular changes

To provide additional vascular context, CD31 (PECAM-1) expression was examined in retinal tissues across the four groups. Western blot showed that CD31 protein levels were significantly elevated in STZ-diabetic retinas relative to non-diabetic controls, with no significant difference between STZ and STZ + sh-NC groups (Supplementary Figure S3A). In the STZ + sh-ANGPTL2 group, CD31 levels were directionally lower than in STZ + sh-NC controls. Immunofluorescence quantification of CD31-positive area showed a consistent pattern (Supplementary Figure S3B). These findings are consistent with elevated endothelial marker expression in diabetic retinal tissues and provide supportive vascular context for the molecular changes observed in integrin α5β1 and PI3K/AKT.

### ANGPTL2 shows protein-level association with integrin α5β1 in overexpressing HRMECs

Co-immunoprecipitation using an anti-ANGPTL2 antibody in OE-ANGPTL2 HRMEC lysates detected integrin α5 and integrin β1 in ANGPTL2 pull-downs but not in IgG control precipitates, supporting a specific protein-level association under overexpression conditions (Supplementary Figure S1B). Co-localization immunofluorescence showed substantial spatial overlap between ANGPTL2 and integrin α5, and between ANGPTL2 and integrin β1 (Supplementary Figure S1A). These findings provide molecular evidence that ANGPTL2 and integrin α5β1 co-exist in close proximity at the protein level under overexpression conditions, consistent with prior reports of ANGPTL2–integrin α5β1 interaction in non-retinal systems. Both assays were conducted under overexpression conditions; endogenous interaction at physiological ANGPTL2 expression levels was not assessed.

## Discussion

The present study shows that ANGPTL2 overexpression promotes multiple angiogenic phenotypes in HRMECs *in vitro*—specifically, enhanced cell viability, wound closure, invasion, and tube formation—accompanied by upregulation of VEGF, p-VEGFR2, integrin α5β1, and PI3K/AKT phosphorylation. These in vitro functional findings constitute the primary contribution of this study and address a gap in the existing literature: the functional consequences of ANGPTL2 dysregulation within retinal endothelial cells had not previously been characterized. The convergent multi-assay functional response to ANGPTL2 overexpression supports the view that ANGPTL2 promotes angiogenic activity in this cell type and is consistent with its established roles in endothelial activation and pathological angiogenesis in other vascular contexts (19). In the context of diabetic retinopathy, the enhanced endothelial viability, migration, invasion, and tube formation elicited by ANGPTL2 overexpression are most plausibly interpreted as pathological rather than reparative angiogenic activity, aligned with vision-threatening neovascularization rather than with beneficial vascular repair or physiological revascularization. This distinction is relevant from vascular-remodeling and regenerative-medicine perspectives, because the same angiogenic machinery can be adaptive during ischemic tissue repair yet detrimental in the diabetic retina; the present findings should be understood in the latter, pathological context. Human tissue-local data reinforce the clinical relevance of these findings: vitreous ANGPTL2 is elevated in patients with active proliferative diabetic retinopathy, consistent with an association between ANGPTL2 and neovascular disease activity (20).

The retinal tissue molecular evidence from STZ-diabetic rats provides supportive background for the *in vitro* findings. Integrin α5β1 expression, PI3K/AKT phosphorylation, and CD31 levels were elevated in diabetic retinal tissues and showed directionally lower levels in the ANGPTL2 knockdown comparison group relative to scrambled controls (Figures 1, 2; Supplementary Figures S2, S3), a pattern consistent with the molecular changes observed in OE-ANGPTL2 HRMECs. The interpretation of these retinal tissue comparisons requires appropriate caution, however. Differences between diabetic and non-diabetic groups arise from a complex hyperglycemic milieu encompassing elevated glucose, oxidative stress, inflammatory signaling, pericyte loss, and basement membrane remodeling—any of which may independently modulate integrin α5β1 expression, CD31 levels, or PI3K/AKT phosphorylation (21). The directional shifts observed in the STZ + sh-ANGPTL2 group are compatible with a partial ANGPTL2 contribution to these molecular changes, but shRNA-mediated knockdown does not uncouple ANGPTL2-specific effects from the broader diabetic tissue environment. Collectively, these findings are best understood as consistent molecular background evidence, rather than *in vivo* functional proof of ANGPTL2 causality. We therefore interpret the retinal tissue data strictly as associative molecular observations within the diabetic retinal environment, rather than as direct functional evidence of ANGPTL2-driven retinal vascular modulation.

The co-upregulation of integrin α5β1 with ANGPTL2 overexpression—observed in HRMEC protein analysis (Figure 4B) and across the retinal tissue comparison groups (Figure 1), and supported by the protein-level association detected by co-immunoprecipitation and co-localization (Supplementary Figure S1)—supports integrin α5β1 as a plausible molecular correlate associated with ANGPTL2-associated angiogenic responses in the retinal endothelial context. Whether integrin α5β1 upregulation functionally mediates the angiogenic phenotypes observed in OE-ANGPTL2 HRMECs was not directly tested in this study. Future studies employing siRNA-mediated knockdown of integrin α5β1 are warranted to determine whether this molecular correlate functionally contributes to ANGPTL2-driven angiogenic responses.

The PI3K/AKT phosphorylation changes accompanying ANGPTL2 overexpression in HRMECs (Figure 5), and the parallel directional pattern in retinal tissue groups (Figure 2), are consistent with broad angiogenic activation and compatible with downstream integrin-linked signaling (22). However, these phosphorylation observations remain correlational in the absence of pharmacological pathway inhibition. PI3K/AKT activation is a shared downstream feature of diverse angiogenic stimuli, and its elevation in OE-ANGPTL2 cells and in diabetic retinal tissues does not demonstrate it as a specific effector axis in this model. These findings provide signaling-associated context that motivates future investigation.

Several limitations of this study warrant acknowledgment; the main observations, the type of evidence supporting each, and the corresponding evidentiary limitations are summarized in Table 2. The functional evidence for ANGPTL2’s angiogenic role rests on *in vitro* overexpression experiments; direct *in vivo* evidence for ANGPTL2-driven retinal vascular functional changes was not obtained. The retinal tissue molecular comparisons are subject to hyperglycemic background confounding, precluding attribution of observed changes solely to ANGPTL2. Specifically, no functional vascular assessments—such as retinal flat-mount vascular density or neovascular tuft quantification, fluorescein angiographic vascular leakage, capillary or pericyte degeneration (e.g., acellular capillary counts on trypsin-digested retinas), or OCT-based retinal morphometry—were performed; the in vivo data are therefore limited to molecular expression comparisons and provide supportive context rather than functional evidence of ANGPTL2-driven retinal vascular pathology. Integrin α5β1 was neither blocked nor knocked down in these experiments, and its causal contribution to the observed angiogenic phenotypes cannot be determined from the current data. PI3K/AKT data are observational in the absence of pharmacological inhibition. The Co-IP assay was performed under overexpression conditions as a single representative experiment; endogenous ANGPTL2–integrin α5β1 interaction at physiological expression levels was not assessed. The STZ model used here represents early-to-intermediate DR, and extrapolation to proliferative disease stages is not supported by current experimental data (23). We further note that alternative models of pathological neovascularization, such as oxygen-induced retinopathy (OIR) and laser-induced choroidal neovascularization (CNV), reproduce retinal or choroidal neovascularization but do not faithfully recapitulate human proliferative diabetic retinopathy; accordingly, no claims regarding proliferative-stage disease are made here, and our conclusions are confined to the early-to-intermediate stage represented by the present model. Experiments were conducted in male rats only, and sex-related variability was not assessed. *In vitro* experiments were conducted under standard culture conditions; the influence of diabetic stimuli such as high glucose, advanced glycation end products, or hypoxia on ANGPTL2-mediated responses was not assessed. The wound healing assay did not include anti-proliferative treatment; therefore, the contribution of proliferation to wound closure cannot be excluded. More broadly, because neither integrin α5β1 nor PI3K/AKT was functionally perturbed in this study, the proposed ANGPTL2–integrin α5β1–PI3K/AKT relationship remains correlative in the present dataset; establishing it will require dedicated loss-of-function experiments—integrin α5β1 siRNA knockdown or function-blocking antibodies, together with selective PI3K/AKT inhibitor or rescue experiments—which we regard as essential next steps before mechanistic or translational conclusions can be drawn. In addition, the retinal immunofluorescence for integrin α5β1 (Figures 1C,D) was not co-stained with an endothelial-specific marker such as CD31 or isolectin B4; the signals therefore reflect whole-tissue rather than endothelial-specific localization, and endothelial-specific co-localization analyses will be needed to confirm the vascular compartment in which these molecular changes occur.

**Table 2:** Summary of major findings and evidentiary limitations.

Figure	Main observation	Type of evidence	Experimental conditions	Evidentiary limitation
Figure 3 (HRMEC functional)	ANGPTL2 overexpression significantly enhanced HRMEC cell viability, wound closure, invasion, and tube formation	In vitro cell-function evidence; CCK-8, wound healing, Transwell, tube formation	In vitro; *n* = 3 independent experiments; ANGPTL2 overexpression model	Strongest functional evidence in this study; overexpression model only; no loss-of-function (siRNA) performed; causal role of integrin α5β1 upregulation not demonstrated
Figure 4 (HRMEC molecular markers)	VEGF, p-VEGFR2, integrin α5, and integrin β1 protein levels increased in OE-ANGPTL2 cells	In vitro protein expression; WB	In vitro; *n* = 3 independent experiments	Molecular correlate; integrin upregulation observed but functional contribution not tested
Figure 5 (HRMEC PI3K/AKT)	p-PI3K/PI3K and p-AKT/AKT ratios significantly elevated in OE-ANGPTL2 HRMECs	In vitro phosphorylation observation; WB	In vitro; *n* = 3 independent experiments	Correlational observation; no pharmacological inhibition; no pathway-level proof of PI3K/AKT as downstream effector
Figure 1 (Retinal integrin α5β1)	Integrin α5 and β1 mRNA and protein elevated in STZ-diabetic retinas; directionally lower in STZ + sh-ANGPTL2 group	Retinal tissue expression comparison; RT-qPCR, WB, IF	In vivo tissue; *n* = 3 biological replicates per group; 12-wk STZ model	Supportive molecular evidence of co-variation with ANGPTL2 levels; no functional integrin blocking or knockdown; no *in vivo* causal relationship demonstrated
Figure 2 (Retinal PI3K/AKT)	p-PI3K/PI3K and p-AKT/AKT ratios elevated in diabetic retinas; directionally lower in STZ + sh-ANGPTL2 group	Retinal tissue phosphorylation comparison; WB	In vivo tissue; *n* = 3 biological replicates per group; 12-wk STZ model	Signaling-associated expression-level observation; no inhibitor experiments; PI3K/AKT not designated downstream effector from these data alone
Supplementary Figure S1 (ANGPTL2–integrin association)	Co-IP detected integrin α5 and β1 in anti-ANGPTL2 pull-downs but not in IgG controls; co-localization IF showed merged signal	Protein–protein interaction under overexpression; Co-IP, co-localization IF	In vitro; OE-ANGPTL2 HRMECs; representative experiment	Protein-level association at protein level under overexpression conditions; endogenous interaction not assessed; no functional blocking performed
Supplementary Figure S2 (Retinal ANGPTL2)	ANGPTL2 mRNA and protein elevated in STZ retinas; reduced by sh-ANGPTL2	Retinal tissue expression; RT-qPCR, WB	In vivo tissue; *n* = 3 biological replicates per group	Verifies ANGPTL2 expression levels and shRNA knockdown efficiency across experimental groups
Supplementary Figure S3 (Retinal CD31)	CD31 protein and IF-positive area elevated in diabetic retinas; directionally lower in STZ + sh-ANGPTL2	Retinal tissue expression; WB, IF	In vivo tissue; *n* = 3 biological replicates per group	Supplementary vascular marker context; no direct assessment of vascular density or permeability

## Conclusion

The present study provides direct *in vitro* evidence that ANGPTL2 overexpression promotes angiogenic responses in HRMECs, including enhanced cell viability, wound closure, invasion, and tube formation, accompanied by upregulation of VEGF, p-VEGFR2, and integrin α5β1. Retinal tissue molecular comparisons across groups with varying ANGPTL2 levels—including integrin α5β1 expression, broader signaling-associated changes, and CD31 expression—together with the ANGPTL2–integrin α5β1 protein-level association detected by co-immunoprecipitation, provide supportive molecular context consistent with potential involvement of ANGPTL2 in the retinal vascular molecular landscape of DR. These findings support further targeted evaluation of the ANGPTL2–integrin α5β1 relationship in retinal endothelial angiogenic responses and provide a rationale for future *in vivo* investigation of ANGPTL2’s contribution to retinal vascular integrity.

## Data Availability

The original contributions presented in the study are included in the article/Supplementary material, further inquiries can be directed to the corresponding author.

## Supplementary material

SUPPLEMENTARY FIGURE S1 ANGPTL2 shows a protein-level association with integrin α5β1 in ANGPTL2-overexpressing HRMECs. **(A)** Co-localization immunofluorescence of ANGPTL2 with integrin α5 and with integrin β1 in OE-ANGPTL2 HRMECs. **(B)** Co-immunoprecipitation in OE-ANGPTL2 HRMEC lysates: anti-ANGPTL2 pull-down probed by western blot for integrin α5 and integrin β1, with normal IgG as the negative control. A representative experiment is shown.

SUPPLEMENTARY FIGURE S2 ANGPTL2 expression in retinal tissues across the four experimental groups. RT-qPCR and western blot analysis of ANGPTL2 in retinal tissues from Control, STZ, STZ + sh-NC and STZ + sh-ANGPTL2 groups (*n* = 3 biological replicates per group, each representing an individual retina), confirming elevated ANGPTL2 expression in diabetic retinas and the efficiency of shRNA-mediated ANGPTL2 knockdown. Data are presented as mean ± SD.

SUPPLEMENTARY FIGURE S3 CD31 expression in retinal tissues across the four experimental groups. **(A)** Western blot and quantification of CD31 normalized to β-actin. **(B)** Immunofluorescence staining for CD31 and quantification of CD31-positive area. *n* = 3 biological replicates per group. Data are presented as mean ± SD.
