# Supplementary material for: ANGPTL2 overexpression promotes angiogenic responses in human retinal microvascular endothelial cells with accompanying integrin α5β1 molecular changes
Source: Front Med (Lausanne). 2026 Aug 3;13:1846631. doi: 10.3389/fmed.2026.1846631 (PMC13478920; doi:10.3389/fmed.2026.1846631)
Supplement: Supplementary file 1 [file Table_1.docx]

Supplementary Table S1 shRNA sequences

| **Number** | **TargetSeq** |
| --- | --- |
| sh-Angptl2-1 | CCATTTGTGTCAACTCCAA |
| sh-Angptl2-2 | GCTTCTACACGAGATCATT |
| sh-Angptl2-3 | GGAGAATACCAACCGCCTT |
| sh-NC | TTCTCCGAACGTGTCACGT |

Supplementary Table S2 Primer sequences of related genes

| **Gene** | **primer** (5'to3') |
| --- | --- |
| Angptl2-rat-F | ATTGCCCAGCTTGAAGAG |
| Angptl2-rat-R | GATGATGCGGTTGTAGGTT |
| actin-rat-F | ACGTTGACATCCGTAAAGAC |
| actin-rat-R | GAAGGTGGACAGTGAGGC |
| integrin alpha 5-rat-F | CTGGGAGGTTTAGGAAGCG |
| integrin alpha 5-rat-R | CGGAGAAGCCAAAGAGGG |
| integrin β1-rat-F | GACGAAAGTGCTCTAACA |
| integrin β1-rat-R | CTGAAGGACCACCTCTAC |
| Angptl2-human-F | TCCCATCTTCCACCGACAA |
| Angptl2-human-R | GCCGTCAATGTTCCCAAA |
| actin-human-F | ACGTGGACATCCGCAAAG |
| actin-human-R | TGGAAGGTGGACAGCGAGGC |
